# Socio-psychological determinants of scabies contact notification among Dutch students: A qualitative study

**DOI:** 10.1371/journal.pntd.0013471

**Published:** 2025-09-04

**Authors:** Sunia B. Somra, Hélène A.C.M. Voeten, Inge Lewis - van Disseldorp, Fraukje E.F. Mevissen

**Affiliations:** 1 Public Health Service Rotterdam Rijnmond, Rotterdam, the Netherlands; 2 Department of Public Health, Erasmus MC, University Medical Center Rotterdam, Rotterdam, the Netherlands; 3 Public Health Service Hollands Midden, Leiden, the Netherlands; Cyprus International University: Uluslararasi Kibris Universitesi, CYPRUS

## Abstract

Recently, the incidence of scabies in the Netherlands increased, especially among young adults. Students are particularly at higher risk of scabies infection, as they often live close together and have many contacts. To prevent ongoing transmission and enhance timely treatment, it is important for scabies patients to notify their contacts. We held in-depth semi-structured interviews with Dutch students who experienced scabies in the past year (n = 15). We investigated to what extent they notify their contacts correctly and timely, and what socio-psychological factors influence their contact notification (CN). Thematic analysis was used to analyze verbatim transcripts. The results showed that most participants seemed to correctly notify their contacts, except for casual bedpartners who were often not contacted or not in time. Individual factors that seemed to influence this behavior were knowledge and awareness regarding CN, perceived risk of transmission, attitude towards CN, emotions involved with CN, quality of relationship with the contact, and intention for CN in the future. Important environmental factors were perceived social norm, received response to notification and disclosure, and experienced stigma. There is a need to develop interventions to enhance CN among students, focusing on knowledge and awareness regarding CN, emotions involved with CN, perceived descriptive norms, and experienced stigma.

## Authors summary

The incidence of scabies is rising among young adults in several European countries. We explored to what extent Dutch students with scabies notify their contacts correctly and timely, and what socio-psychological factors influence their contact notification (CN). Our findings indicate that most participants seemed to correctly notify their contacts, except for casual bedpartners who were often not contacted or not in time. Participants reported that the guidelines for notification were unclear. They also expressed uncertainty about how they became infected and the effectiveness of the treatment (Knowledge and Awareness regarding CN). Additionally, negative emotions sometimes prevented them from notifying promptly or at all (Emotions related to CN). While participants believed that their peers were likely to notify their contacts, they also mentioned several reasons that might prevent them from doing so (Perceived social norm). Furthermore, participants recognized that there is a stigma surrounding scabies among individuals outside their student community (Experienced stigma). To encourage students with scabies to notify their contacts, future interventions should address the behavioral factors mentioned above. For example, by stimulating students to think about the consequences of not notifying their casual bedpartners, or by addressing stigmatizing perceptions of individuals outside of the student circle.

## Introduction

Scabies is an infectious disease caused by Sarcoptes scabiei var. hominis mite, which manifests itself with symptoms such as intense itching, skin lesions and burrows. The persistent itch drives people to scratching, which can disrupt the skin barrier and may result in secondary bacterial infections. Scabies spreads directly through prolonged skin-to-skin contact and indirectly via clothing and bedding [1,2]. The treatment options for scabies include permethrin cream and ivermectin pills [3,4]. In the Netherlands, there is a rise in the incidence of scabies among young adults [5]. Especially students are at high risk of contracting scabies, due to living close together in student houses and regular sharing of clothes and bed [6]. There is no clear explanation for the rise in scabies within this age group.

One of the crucial aspects of preventing further spread of scabies is contact notification (CN). CN involves notifying individuals who may have been exposed to scabies so that these contacts can take preventive measures [7]. These include preventive treatment with permethrin cream or ivermectin pills, washing bedding and clothes at 60°C, and sealing items that can’t be washed in a plastic bag for a week [3,4,8]. According to the Dutch national guidelines for scabies [8], contacts that should be notified include roommates, sexual partners, and people with whom one had skin-to-skin contact for more than 15 minutes, or with whom one shared bed, clothing, or towel in the past 6 weeks prior to knowing one’s scabies status. Accurate CN can help prevent further spread of the (re)infections [8]. To what extent Dutch students notify their contacts completely and timely is unclear. However, research on CN for other diseases such as sexually transmitted infections (STIs) suggests that this behavior is not always performed correctly and depends on several factors inhibiting or facilitating the behavior [9–12]. Hence, it is important to understand the various social and psychological factors that influence CN, to inform future interventions promoting CN for scabies among students.

Behavior is determined by both individual factors and the interaction with the social environment [13,14]. To our knowledge, no previous studies have been published so far on the factors that influence CN for scabies. However, the determinants of CN are well studied for other diseases like STIs [9,10]. Individual determinants that influence CN for STIs are knowledge regarding CN, awareness regarding CN, perceived risk for transmission, attitude towards performing CN, emotions involved with CN, intention for CN in future, and self-efficacy regarding CN [15–21]. A study on partner referral among women with chlamydia, for example, showed that emotions, such as fear of negative reactions, played a significant role in the decision to not notify their contacts [17,18]. Also, individuals with a positive attitude towards CN and who acknowledge its importance have stronger intentions to carry out CN [15,16,22]. Environmental determinants such as perceived descriptive norm, perceived injunctive norm, experience stigma, reaction of others to CN, and social support for performing CN have an impact on CN for STIs [9,23–25]. A study by El-Sadr et al. found that stigma associated with STIs often acts as a major obstacle to CN [9]. As scabies is a visible skin disease often incorrectly associated with inadequate hygiene, it seems likely that it may evoke stigma as well [26]. Finally, individuals adapt their behavior according to the supposed CN actions of the majority of the group (descriptive norm), but are also sensitive to what others expect them to do (injunctive norm) [23]. In addition to empirical findings, further support for the above-mentioned determinants in influencing behavior is provided by health behavior theories such as the socio-ecological model, the Theory of Planned Behavior and the Reasoned Action Approach [27–29].

This qualitative research aims to explore to what extent students with scabies notify their contacts completely and timely, and which socio-psychological determinants may influence this behavior. Insight into these factors can be used to design interventions to improve CN and ultimately prevent further (re)infections [30].

## Methods

### Ethics statement

Since participants were not subject to procedures or were not required to follow rules of behavior, ethical approval was waived by the Medical Ethics Review Committee at Erasmus MC, University Medical Centre Rotterdam (MEC-2023–0259).

### Study design

In-depth semi-structured interviews were performed with students who experienced scabies in the year prior to the interviews. Since little is known about socio-psychological determinants that play a role in CN among individuals with scabies, the use of a qualitative research design provided the flexibility to ask follow-up questions to better understand complex situations [31], and gain deeper insights in the socio-psychological determinants. The qualitative interviews supplemented an online survey study on the same topic which preceded the current study.

### Participant recruitment

Students for this study were recruited via an online survey on scabies experiences among students in the Netherlands. Towards the end of that survey, it was stated that students who were interested could sign up for a qualitative follow-up study (interview) on scabies. It was mentioned that participation in this follow-up study was voluntary, confidential, and that a compensation of 25€ would be provided. Those interested in joining could enter their e-mail address.

**Fig 1:**
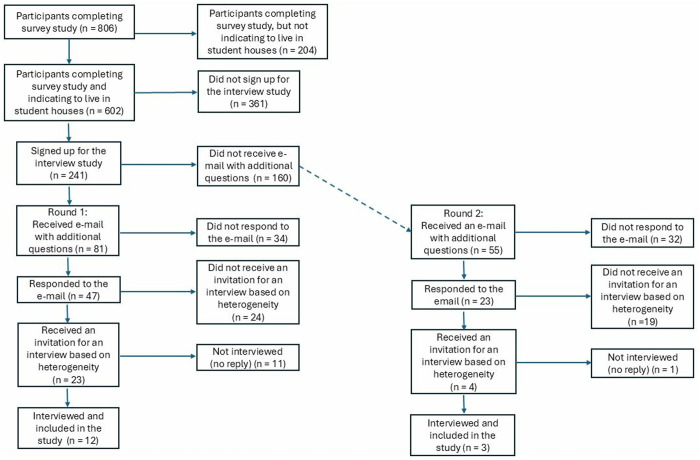
Participant Sampling Flowchart.

Fig 1 illustrates the process of the recruitment and participant sampling. In total, 602 students living in student houses or similar settings who filled in the survey received this invitation of whom 241 (40%) signed up for our follow-up study. Every third student who signed up (n = 81, 34%) received an e-mail in which they were thanked for their interest and asked to reply to some questions by e-mail in order to collect information on their gender, the city where they were studying, whether they experienced scabies, and when their most recent scabies infection had occurred. This information was used for purposeful sampling: it was our aim to include a heterogenous sample in our study with regard to gender and city of study. In addition, students who experienced scabies more than one year prior to the interview were excluded from this study. Forty-seven students (58%) responded to these questions via e-mail, of whom 23 (49%) were sampled based on heterogeneity and eligibility. They were invited via e-mail to participate in the interview. In the end, twelve participants responded to this invitation and were interviewed at a day and time according to their preference.

We were close to data saturation after these twelve interviews, but a few additional interviews were scheduled to verify [32]. To this end, the same recruitment procedure was applied to the 160 students who had not yet received an invitation (n = 241 – 81). In total, 55 additional students were invited in this second round, of whom 23 students (42%) responded to the e-mail. In the end, three students were interviewed based on our heterogeneity and eligibility criteria. After these three additional interviews, data saturation was confirmed.

### Study participants

A total of 15 students participated, with a mean age of 21.4 years (SD = 1.8, range 19–24). Eight were female and seven were male. Five participants studied in Rotterdam, five in Leiden, three in Utrecht, and one each in Delft and Enschede. All participants lived in student houses, or a comparable accommodation, and they lived on average with eight other roommates (SD = 5.8, range 3–19).

### Study procedure

Prior to the interview the students were informed about their privacy and their rights as participants in this study. They were assured that participation was voluntary, and that they had the right to refuse to answer questions if they did not feel comfortable. Additionally, they were informed that their answers would be handled anonymously, that no demographic data would be asked or noted during the interview, and that the interview would be recorded and stored securely and deleted after the interview had been transcribed. The first six interviews were conducted by a senior and a junior researcher to guide the primary (junior) interviewer and to perform a quality check on the interviews. The remaining nine interviews were conducted by the primary researcher. The average duration of the interviews was 74 minutes (range 41–120 minutes). All the interviews were conducted in Dutch and online via Microsoft Teams. We chose for online interviews in order to facilitate the inclusion of participants from all across the Netherlands. Also, online interviews are more convenient for students and still produce good data quality [33]. To limit the impact of external distractions [33] students received clear instructions by e-mail on selecting a quiet and private space for the interview. To provide sufficient empathy, each interview started with a warm-up talk to make the participant feel more comfortable. After each interview, notes were made on the course of the interview to capture the non-verbal cues and emotions of the participant [33].

### Interview protocol

The interview protocol was developed based on a review of the literature and informed by theories of health behavior [27–29]. The interview protocol was structured thematically covering the following topics: background information (including age, gender, and living conditions), experience with scabies (including emotions and people’s responses), and experience with and performance of CN (i.e., who was notified, when and how, the criteria they applied for notifying someone, their opinion on CN, need for support, experienced stigma, and norms surrounding CN). Finally, we asked how they thought CN among students could be promoted. The main focus of the interview was on the most recent scabies episodes, however previous scabies episodes were also discussed. The participants’ experience of scabies was self-reported, and we did not probe whether they consulted a GP for getting a scabies diagnosis.

### Data analysis

All 15 interviews were audio recorded after which they were transcribed verbatim. Atlas-ti software version 23 was used to analyze the data. Thematic analysis was used to analyze the transcripts [34]. The researcher’s background, assumptions, and perception can influence the interpretation of the transcript and the results. However, to minimize the researchers’ bias, we chose to review each step of the analysis with multiple researchers. The first three interviews were individually coded by two researchers, after which discrepancies were discussed until agreement was reached. Then the remaining interviews were coded by the primary researcher. Hereafter, the codes were categorized into themes and summarized by the primary researcher. Two senior researchers reviewed the themes and summaries, after which disagreements were discussed with the primary researcher until consensus was reached. The notes taken after each interview were used to provide a general impression in the results section on data quality and non-verbal cues.

## Results

Most participants expressed an eagerness (both verbally and non-verbally) to talk, resulting in relatively long interviews (average duration of 74 minutes). They were very open about their experience with CN and were happy that they were able to share their story with someone. Moreover, they talked about their experience with scabies and elaborated on the severity of their symptoms and the treatment they underwent. The participants did not always consult a GP for getting a diagnosis when experiencing scabies-related symptoms. Instead, they often relied on their own previous experiences or those of their peers. Although the treatment for scabies can be expensive, the costs did not appear to be a concern for them. We do not elaborate further on these findings as it is beyond this paper’s scope; they are discussed in more detail in another study.

**Fig 2:**
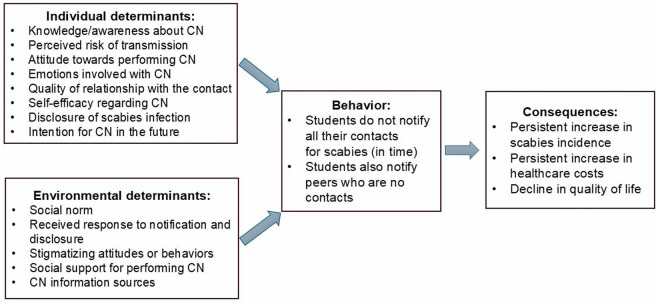
Conceptual model of CN behavior.

In this results section, first, participants’ CN behavior will be described, followed by individual determinants and environmental determinants related to CN. Individual determinants include knowledge and awareness regarding CN, perceived risk of transmission, attitude towards performing CN, emotions involved with CN, quality of relationship with the contact, self-efficacy regarding CN, disclosure of scabies infection, and CN intention in the future. Environmental determinants include perceived social norm, received response to notification and disclosure, experienced stigma, social support for performing CN, and CN information. Fig 2 illustrates the conceptual model of CN behavior based on our results. The most illustrative quotes for each determinant are mentioned in the text; additional quotes can be found in Table 1.

**Table 1:** Overview of socio-psychological determinants and associated quotes.

Themes	Subthemes	Respon-dent	Quote
CN behavior		1	Yes, the first time I notified everyone. The second time, I also notified everyone, but there were fewer people. It was only... one person. So, it was much easier.
		4	No, I actually only... for example, my sister, who graduated, so she hasn’t been as familiar with it [scabies]. But the people who are still in student life, I know that everyone is generally aware of how it works and can look it up if needed, at least the people in my circle. So, I just indicated to them that if I were them, I would also treat it and didn’t provide any further guidelines. But, for example, I did tell my sister, “Well, it’s clearly stated here, so definitely follow it.”
Individual determinants	Knowledge/ awareness about CN	11	…But this was for example already after the second treatment, so I thought, “Oh, well, it should be fine now.” […] So, I was also a bit like, “Well, it should be ok now,” but I wasn’t sure because sometimes it turned out that the cream didn’t help.
	Perceived risk of transmission	5	Look, during the day you try to keep some distance and be cautious, but for example, in the evening, at a party, if you’re lying together on the couch or something, you don’t really pay as much attention to it as you do during the day in the living room. (…) …but in the evening, you let that slide a bit when you’ve had a few beers.
		12	...and after 24 hours, you are temporarily safe.
	Attitude towards performing CN	8	Interviewer: “Yes, did you find it difficult to tell people that you have scabies?” Respondent: “Yes, well, the first time I really did [find it difficult], because I didn’t really know much about it yet. But the second and third times, not at all. Yes, it still feels dirty and difficult, but I thought I just needed to do it, and it’s better to do it as soon as possible. Yes, I learned that from the first time.”
	Emotions involved with CN	3	And at that moment I was also dating someone, so I also needed to notify him. That is also a bit uneasy…
		8	Yes. Yes, I really noticed that it felt like a weight was lifted off my shoulders once I had told my parents and my housemate and had talked to them about it, and it turned out that it wasn’t as bad as I had thought.
	Quality of relationship with the contact	13	It’s just natural, you talk about everything with your housemates every day, so it’s less awkward to discuss stupid things as well. Whereas if you see someone less often, or something like that, yeah, then sometimes you’ve never even texted with someone, and then it’s not exactly nice for the first text to be, ‘Hi, I have scabies’.
	Self-efficacy regarding CN	7	Interviewer 1: “Yes. Do you trust that you’ve informed everyone, or yeah, the right person?” Respondent: “Yeah, I think so. I didn’t give anyone scabies.”
	Disclosure of scabies infection	2	Well, I also didn’t tell the people who didn’t needed to know.
	Intention for CN in the future	7	Interviewer: “…Um, imagine that in a few months you have scabies again. Would you handle the contact notification in the same way?” Respondent: “Yes, I think so, but I keep hearing you talk about the guidelines, so I might look those up.”
Environmental determinants	Perceived social norm	6	Well, I know those are just stories, but for some people it really is difficult to tell someone they only spent one night with and then never see again that they have scabies…
		4	Um, in general, my housemates expect that if someone — not just me — has scabies or is affected by it, they should be honest about it, so we can find a solution together and help each other. I think people you’ve shared a bed with would also expect that, but they might be less likely to say it out loud.
		13	My immediate surroundings, like my home and family, yes, but outside of that, I don’t know, because I have, well, as I said, a lot of people didn’t do it for me either, so I don’t think they expect you to return the favor or anything.“
	Received response to notification and disclosure	13	And in the end, even though getting the message isn’t pleasant, you’re ultimately glad that someone told you. That way, you can do something about it yourself.
		6	People tend to take it very personally, and then I think, oh yes, well, I… I lie awake at night [from the itching], this and that, you know. So, I think the worst reactions are those that don’t recognize the mental issues. I believe those are the worst reactions. But that’s not a direct reaction; it’s an indirect response to it.
		5	Well, for example, people might avoid sitting next to you on the couch, those kinds of things. You definitely notice that people start to take exaggerated steps to keep their distance, even though if you’re sitting at a reasonable distance on a leather couch, there’s no need to be overly concerned. So, it’s things like that, where you can tell people are taking it very seriously. Yes, you definitely notice it.
	Experienced stigma	6	I know, we talked about this with my parents, and they felt it was a bit more awkward, you know, in the adult world — what am I even saying — but, like, in their circles, they’re just, they’re 50-plus, it’s really more of an issue of ‘You have scabies? How do you have scabies? You know, what are you doing?’ But in student circles, you can literally walk in and say to everyone, ‘Yeah, I’ve got scabies,’ and it’s really not a problem.
	Social support for performing CN	1	Uhm no, no I didn’t have the feeling that that [support] was necessary, but that of course also depends on the environment. Yes, I have lovely friends, so I was not afraid that they would react strangely, so in that respect no, no.
	CN information sources	1	Well, the RIVM [the institute publishing the national guidelines] simply said that yes, you have to do all that, but it is not that the GP... Yes, the GP did say that the housemates and bedpartners should also treat, but it’s not that someone said, “you should really call him now, because it’s sad for him or something.

### CN behavior

The participants gave the impression to mostly have notified the correct contacts, according to Dutch national guidelines [8], which often included housemates, family, friends, guests (including sleepovers), and (bed)partners.

*“Well, first of all, I informed all my housemates. Then, um, actually all the friends here in Leiden that I had seen recently. I also sent a general message to the group saying, “Hey guys, just keep an eye on this.” Um, yeah, also, a friend of mine who had stayed over at my place, I gave him a call. Basically, everyone I had seen in the past few days with whom I had close contact with, I either called or sent a message.” (Respondent 9)*

However, less closely related contacts, such as one-night stands, fellow students, or people considered less at-risk were sometimes informed a few days later or only when they met by coincidence. Contacts were mostly notified in case the last moment of contact was within the past two weeks to one month prior to the moment scabies appeared or was diagnosed. Having scabies symptoms or having it officially diagnosed by a doctor was often the moment people started to trace and inform their contacts. Most participants used WhatsApp to inform their contacts, citing its speed and practicality especially when large groups (e.g., housemates) needed to be reached. The majority stated they had advised their contacts to take preventive measures or treatment. However, the level of details added to this general advice varied and sometimes seemed to depend on the type of contact.

Half of the participants had not received any scabies contact notification themselves prior to their scabies experience and had no clue how they contracted scabies or had only a vague idea (via couch, clothes etc.). If participants received a CN, this was often indirectly by, e.g., their housemates or partners, but not by the source of their infection. Not having received a CN was sometimes stated as a reason for not performing CN.

### Individual determinants influencing CN

#### Knowledge and awareness regarding CN.

All participants were aware of the need to inform their contacts, either because they were notified themselves or because of information they obtained online or via others. The level of specific knowledge on transmission risks and who is defined as a contact was quite detailed among many participants: they mentioned bedpartners and people with whom you shared clothes and/or with whom you had physical contact for at least 15 minutes as contacts to be at risk. On the other hand, their responses showed that many aspects were still unclear, such as up to how long ago do contacts need to be notified, whether the fabric of furniture plays a role in the risk of transmission (leather, plastic, cotton), and whether you are still infectious after treatment.

*“When you’re in a student association, you know so many people. It’s very difficult to distinguish who your contacts are and who not. But I think that the people I see daily and who are close to me, I consider them as contacts.” (Respondent 5)*

#### Perceived risk of transmission.

Although participants did not directly state how big they estimated the risk of having infected others with scabies, this can be inferred indirectly from their comments. The participants (almost unanimously) estimated the risk of transmission to be highest when sharing fabric sofas, clothing, towels, or beds. Moreover, the participants mentioned that the following contacts were at risk of infection: individuals with whom they had more than 15 minutes of skin-to-skin contact, people living in the same household, (bed)partners, regular visitors, and others close to them.

At the same time, however, there were also several contradictions or uncertainties regarding what was perceived as a risk or not, such as the 15-minute skin-to-skin contact (is hugging risky or not?) and the shared use of sofas, with varying ideas on when this is risky or not, depending on the materials and the exact position taken on a shared couch. It seems participants often relied on intuition to assess the risk, even when aware of the existing guidelines. They, for example, perceived the risk to be low when, in their opinion, they had taken good preventive care.

*“And, um, I did look up the RIVM guidelines, but I have to say that I didn’t really follow them strictly; instead, I relied more on my intuition and assessed for myself how likely it was that I might have infected someone.” (Respondent 8)*

Most respondents also perceived a risk of transmission after their (full) treatment because treatment is not always successful, and one might still be infectious. They therefore avoided possible risks by not sitting on the couch for example and notifying their contacts again.

*“…Then, um, because after that first application, I thought... I know that my boyfriend also wasn’t free of it [scabies] after the application. So, just to be sure, I didn’t sit on the couches or any fabric... I only sat on wooden surfaces and in my own room, not on the couch.” (Respondent 10)*

#### Attitude towards performing CN.

All participants acknowledged the importance of CN in preventing the further spread of scabies and potential reinfection. However, they found it sometimes difficult to do so consistently.

*“Yes, yes, yes, I personally don’t find it difficult [to notify] for housemates. I don’t find it difficult with my parents either, but it’s more about people you’ve slept with. That’s where things mostly go wrong, I think. It’s something people find harder to talk about, and I do too.” (Respondent 13)*

The feasibility of contact notification depended on the type of contact and whether the person being contacted was being notified for the first time by the participant. Some participants found it harder to inform others about scabies the first time than during subsequent infections. This was because the first time they were concerned about other people’s reactions, or that their familiarity with scabies was limited. Others found it more difficult during subsequent infections, as they had to bother others again about a possible infection (and the preventive measures they had to take). Participants acknowledged the importance of knowing whom you contracted scabies from, as it helps in, for example, how far back in time you need to identify your contacts.

#### Emotions involved with CN.

All participants expressed negative emotions when reflecting on their contact tracing experience, although these feelings were followed by relief afterwards when the contact’s response turned out not to be bad. “Guilty” was the emotion most often used by the students to describe their feelings, because ”you burden someone with a lot of work”.

*“Um, I think I felt guilty, because I was afraid, I might pass it on to someone else, rather than feeling ashamed, because I know it’s not something I can control. And that, that’s what I felt most; guilt, because I was afraid, I might burden someone else with it.” (Respondent 4)*

Other emotions mentioned were feeling annoyed, feeling anxious or being worried about the other person’s reaction or about gossiping, feeling shame (e.g., because it is the umpteenth time), or feeling uncomfortable.

The emotions partly depended on the relationship with the contact. Emotions like worry were more prominent with casual bedpartners, acquaintances, or new partners, because it is more difficult to anticipate their response or because of fear of rejection. These emotions sometimes caused a delay in the notification or, in the case of one-night stands, in not notifying at all.

#### Quality of relationship with the contact.

The quality of the relationship with the contact sometimes determined whether the contact was notified. This was true especially for one-time bedpartners. Some participants stated that their decision on alerting somebody was related to a person being a good friend or being nice.

*“Well, there was one guy I saw only once. Then I was unsure, I’m never going to see him again, so I thought, well, maybe I don’t need to tell him. But I did like him, so I thought, yeah. And I would want to know myself too, so I think I ended up waiting almost 4 days before telling him.” (Respondent 1)*

#### Self-efficacy regarding CN.

Most participants were confident or even very convinced that they had been able to trace down all their contacts correctly. Moreover, they were confident that their approach to notify their contacts was effective. Their confidence was based on arguments such as: the decision to notify someone was well thought through; they could freely talk about their scabies infection; the conviction to not have infected somebody; or they had contacted nearly everybody in their network. Previous CN experience was also used as an argument to support their self-efficacy.

*“I just felt that I had handled it well myself.” (Respondent 7)*

#### Disclosure of scabies infection.

Most participants seemed to be quite open about their scabies-status and disclosed it also to people who were not necessarily contacts who had to be traced.

*“Well, in that sense, my group of friends wouldn’t need to treat themselves either, because it said you would need to have had skin contact for 15 minutes or if people had slept in your bed, but that’s not the case with them. I still did let them know, also because we had something planned. I thought, well, guys, I think it’s just polite to let you know. If anyone feels uncomfortable about it, then I won’t come, but I’ve already applied the treatment, so it should be fine.” (Respondent 3)*

Participants could openly talk about scabies and make jokes like making a “scabies counter*”* (i.e., counting the number of times someone had experienced scabies) on the wall. Parents or other family members, (close) friends, or people they were visiting were among the people to whom they disclosed their scabies infection. Reasons for disclosing were to enable others to take preventive measures (to prevent transmission), having a close relationship with the person, or more spontaneous when it happened to come up in a conversation. At the same time, participants also stated that they did not announce their status to just anybody, when it seemed unnecessary. In some situations, the participants had to disclose their status (e.g., when avoiding a handshake). Only a few were more hesitant or had delayed disclosure, partly for fear of stigmatizing responses.

#### Intention for CN in the future.

All participants intent to notify their contacts when they would get scabies again, and almost all participants indicated that they would do so in a similar way as they did before. Some participants added that they would first check (again) the guidelines on contact tracing, that the next time they would add less apologies, or that their approach would depend on the situation or person.

*“Yes, I think so, but it also depends on… I don’t know. It’s complicated because it really depends on the situation, who is involved and what’s going on. But I think with my housemates and my parents, I would always tell them. Outside of that, it really depends on the situation.” (Respondent 13)*

### Environmental determinants influencing CN

#### Perceived social norm.

Participants had varying opinions about how well others notify all their contacts (descriptive norm). Most seemed positive and believed that others notify quite well, particularly their friends and people in their network. Other participants were more skeptical and felt that especially parents and casual bedpartners were not always informed.

Few participants found it difficult to estimate how well others notify their contacts. Feelings of shame, fear of stigma and personal experiences of not receiving notifications themselves were cited as reasons why they expected that their peers did not notify all their contacts adequately. They also believed that their peers found it annoying to have to inform others, especially when they had to do so repeatedly because of reoccurring scabies. Selfishness was another argument they mentioned, as students may not feel any personal gain from notifying others, or they may simply feel that it’s not their problem whether contacts avoid getting scabies or not.

*“…I’ve heard stories where people think, oh well, they won’t find out for another eight weeks anyway, so I just won’t tell them… “(Respondent 14)*

Moreover, people whom you don’t see again are easily ignored, because there is no risk of re-infection. The participants mentioned that their peers feel it is too much trouble or they are lax. Some also mentioned that for students, tracing their contacts might not be as much a priority as the treatment itself.

Participants unanimously stated that people in their close social network (i.e., housemates, close friends, bedpartners, and family) would expect to be notified by them in case they would have scabies (injunctive norm), although it may depend on the type of contact.

*Interviewer:” Yes, and who does expect from you that you tell them?”*

*Respondent: “Well, definitely my housemates, uhm friends, yes people whom I slept with, my friends. Especially, when you are somewhere and you have scabies, you tell them “I have scabies, so I cannot sit on the sofa” …” (Respondent 7)*

#### Received response to notification and disclosure.

When asked about how people responded to their scabies infection, it was not always clear whether the participants described people’s responses to their scabies notification or people’s responses to their scabies disclosure. In general, most participants mentioned that the responses were considerate, especially those of other students and housemates they notified. Their peers were understanding and reacted calmly, as scabies is considered “normal” among students. The participants received supporting and sympathetic reactions like “too bad for you”, “it can happen to anyone”, and “it is not your fault”.

*“But no, it really felt like a weight off my shoulders when I told them, and everyone reacted normally, thankfully, and hoped it would go away as quickly as possible.” (Respondent 8)*

However, some students received less positive reactions, such as people responding startled, grumpy, annoyed, looking at you like you are dirty and keeping extreme distance. Or they reacted just with a lack of empathy, particularly people outside of the student circle that were less familiar with scabies, such as parents, family, and colleagues. The participants often stated they understood these (stigmatizing, selfish) reactions. One participant explicitly stated the responses were sometimes quite selfish (i.e., the contacts were thinking about themselves and the actions they had to take to prevent scabies infection) and did not sympathize with the burden for the participant of having sleepless nights.

#### Experienced stigma.

Most students did not feel stigmatized themselves, but they assumed that their peers may feel stigmatized. However, in between the lines a fear for stigma was apparent, and sometimes stigma also seemed to have been experienced. For instance, respondents stated that scabies is often perceived by others as “dirty”, your own fault, and related to (casual) sex. They also mentioned that scabies is sometimes wrongly perceived as an STI, which can make people hesitant to talk about it. However, according to them, stigmatizing responses mostly come from people outside the student world and not from peers, because scabies among students is quite normal nowadays. Finally, several students would welcome stigma reduction; more acceptance that scabies can happen to anyone and more familiarity with scabies may help in improving CN.

*“If the stigma around STI lessens a bit, I think it will also be easier for people to notify others [for scabies].” (Respondent 7)*

#### Social support for performing CN.

The majority of students stated that they did not need (and had not needed) any social support when informing others about their scabies infection. They did come with some suggestions for support, but this was often followed by statements like *“*I don’t need it but maybe others do*”* or *“*I’m not sure*”*. Two participants mentioned that they sometimes discussed with their friends or roommates if and how to notify contacts.

*“I think I always knew who I needed to message, but I usually discussed it with my housemates, like, ‘Okay, guys, who should I inform, how should I handle this, and what exactly should I say?*”* (Respondent 13)*

#### CN information sources.

Participants used various sources to gain knowledge about CN, such as the guidelines from the National Institute for Public Health and the Environment, and www.thuisarts.nl, an independent public website with reliable and accessible information about health and disease created by general practitioners and medical specialists. However, according to some participants, there is a need for clear and comprehensive information about who should be notified and who not, and how far back in time contacts should be informed. For others, the information is clear. Moreover, several respondents mentioned that CN was only briefly discussed during the GP consultation or that the GP did not emphasize the importance of CN enough. Several participants stated that although most people are aware of the need to notify contacts, there should be more emphasis on the urgency of CN.

*“Clear guidelines: Do you need to notify people up to 6 weeks back? Do you need to notify people up to a week back? And out of all those people, who exactly should you notify?” (Respondent 12)*

## Discussion

The aim of this study was to evaluate CN behavior among students with scabies and to gain in-depth knowledge on important determinants that may influence CN. In general, participants seemed to notify most of their contacts in line with the Dutch national guidelines [8]. However, less close contacts such as casual bedpartners were often not contacted or not in time. Our data show that CN seems to be influenced by individual determinants such as knowledge and awareness regarding CN, perceived risk of transmission, attitude towards CN, emotions involved with CN, the quality of the relationship with the contact, self-efficacy regarding CN, and disclosure of scabies infection. Relevant environmental determinants were perceived social norm, received response to notification and disclosure, stigmatizing response, and social support for performing CN. Intentions for performing CN for future scabies episodes were high, but a need for better and more clear information was expressed.

The students in our study sample did not always notify all their contacts or not in time. At the same time, they often tend to notify persons for preventing them to get infected, regardless of whether they have been in close contact or not. This could lead to missing out on individuals who should have been notified and treated or to overtreatment of persons who are actually not a contact. This behavior seems to be partly related to the guidelines not being clear for them, uncertainty on when and how exactly they got infected, and uncertainty on the effectiveness of the treatment which results in constant fear for transmission and continuous CN. There is a strong belief among the student population that fabric furniture contributes to scabies transmission, even though there is no strong scientific evidence on any indirect transmission of scabies [2]. A qualitative study by Trettin and colleagues [35] focusing on the experience of individuals with scabies, also found that participants struggle with whether scabies is effectively treated or not. This often results in feelings of uncertainty and powerlessness. Studies have shown that well written information, for example on a website, can help to improve health behavior [36]. Therefore, existing guidelines on CN should address these uncertainties and emphasize the importance of CN.

Interestingly, our study suggests that the quality of the relationship with the contact influences the decision to notify; contacts who were positively evaluated (friendly, nice, or with whom one had stronger emotional ties) were more likely to be notified. Quality of a relationship refers to how individuals perceive their relationship (i.e., negative, or positive) [37]. Studies on CN by adolescents and adults with STIs also found a positive association between the quality of relationship with the sexual partner and the willingness to notify that partner [38,39]. From an intervention perspective, stimulating CN by directly influencing relationship quality will be difficult, as several factors determine it [37]. More research is needed on how to target the impact of relationship quality on contact notification.

Despite that our participants were conscious about the importance of scabies CN, sometimes emotions resulted in not performing CN or delaying to notify casual bedpartners. Similarly, individuals with STIs often experience emotions like guilt when notifying their sexual partner, which can lead to not notifying at all [7,17]. Negative feelings are especially prominent in causal bedpartners compared to committed partners [40]. Even though people may have negative feelings toward CN, the participants in our study described a feeling of relief after CN. This is why it’s important to develop interventions that encourage positive emotions surrounding CN of bedpartners. This can be achieved by, e.g., using environmental re-evaluation, which means encouraging combining both affective and cognitive assessments of how the presence or absence of a personal behavior affects one’s social environment [41]. In line with this, students can be stimulated to think about the consequences (positive and negative) of not notifying their casual bedpartners and hereby stimulating empathy and encouraging the preferred behavior [42,43].

Disclosure of scabies was high among our participants. When comparing individuals with scabies to those with STIs, the latter tend to be less open about their status [44,45]. The model of health disclosure decision-making could provide explanation for this difference [45]. According to this model when individuals decide to disclose their STI-status, they first assess the information related to their diagnosis (e.g., presence of visible symptoms or stigma), anticipated responses, their efficacy in disclosing, and the potential outcome of their disclosure [45,46]. Anticipated stigma might be an important factor explaining the difference between scabies and STI, as individuals with STI’s fear rejection when disclosing their status to others [45,47,48], which was not evident in our study. The high disclosure rates in our study are a positive outcome, as disclosure has shown to be positively related to engagement in healthy behavior [49,50].

Generally, participants in our study mentioned that they hardly experienced stigmatizing responses during CN, especially among their peers. In contrary, the study of Trettin et al. [35] investigating the experience of individuals with scabies found that the participants felt stigmatized when disclosing their scabies diagnosis The low experienced stigma among the students in our sample might be explained by scabies being “normalized” due to its high prevalence among students. Our participants mentioned that parents and people outside the student circle, where the scabies prevalence is low, can perceive scabies as “dirty” and associated it with sex. Stigmatizing perceptions of individuals outside the student circle can be addressed by using stereotype-inconsistent information and including several role models [51,52] in mass media, such as television campaigns or social media [53]. There should be an emphasis on scabies as something that anyone can get, regardless of hygiene.

Although the participants in our study mostly seemed to think that their peers do notify their contacts, some believed that CN is not always performed by their peers. Studies have shown that descriptive norm has a significant effect on health behavior [54,55]. Intervening in descriptive norm can be valuable to increase engagement in CN. A study by Zhang et al., [54] found that when promoting public health behavior, there should be an emphasis on the existing positive behavior (e.g., the majority of students do perform CN). Moreover, individuals tend to follow the behavior of those close to them [54]. Thus, combining these factors can result in an effective intervention.

Our study had some limitations. Firstly, our sample may be biased, as individuals who are more engaged in CN could have been more willing to participate. Since only half of our participants received a CN themselves, but reported to mostly perform CN, it is well possible that our study sketches a too positive picture of CN behavior and its factors, limiting the generalizability of our results. However, as our study is among the first to thoroughly study determinants of CN for scabies, we believe our results still provide an important contribution to the scientific literature. Another limitation is that the scabies experience was self-reported, and we did not probe whether a GP was consulted. However, we have hardly any doubt about their actual scabies infection as the participants gave detailed information about their symptoms and treatment. Another possible limitation of our study can be the received compensation of 25€ which can unfairly influence or pressure potential participants and compromise their informed consent [56]. However, the participants seemed eager and open to discussing their experiences. Moreover, they did not view the costs of the medication as a significant issue, which can be expensive. Therefore, the influence of compensation, if any, is likely to be small. Lastly, during the interviews we tried to capture information about both disclosure of scabies and about CN. Only during analyses, it became clear that our way of questioning makes it sometimes unsure whether the respondents reflect on their experiences during CN or their experiences during disclosure. For future studies, the interviewer should make it more explicit to what behavior (CN or disclosure) the question relates.

To our knowledge, this study is the first to investigate the role of socio-psychological determinants in CN among students with scabies. By conducting in-depth interviews, we were able to identify individual and environmental factors that play a role in CN. Future studies should focus on developing interventions to enhance CN among students focusing on knowledge and awareness regarding CN, emotions involved with CN, perceived descriptive norms, and experienced stigma.

## Supporting information

S1 File Interview guide for students with scabies. (DOCX)
