# Supplementary material for: Socio-psychological determinants of scabies contact notification among Dutch students: A qualitative study
Source: PLoS Negl Trop Dis. 2025 Sep 4;19(9):e0013471. doi: 10.1371/journal.pntd.0013471 (PMC12410757; doi:10.1371/journal.pntd.0013471)
Supplement: S1 File — (DOCX) [file pntd.0013471.s001.docx]

**S1 File. Interview guide for students with scabies**

**Before starting the interview**

*Thank you and introduction*

My name is (name of the researcher), and I work as a researcher at the Public Health Service Rotterdam-Rijnmond. I want to thank you very much for completing our online questionnaire about scabies and for your willingness to participate in this interview.

*A brief explanation of the research*

There has been a significant increase in the number of scabies infections among students. You have also experienced this yourself. The treatment of scabies is often a long process during which many reinfections occur. Together with the Public Health Service Hollands Midden and the Erasmus MC, we are conducting research into the experiences of students with scabies and the actions they take when infected with the scabies mite. Based on our findings, we would like to offer recommendations on how the information provided to students can be improved to combat scabies more effectively.

*Information about participation and the interview*

Participation in this research is voluntary. You are not obligated to answer all questions. The interview is anonymous; no personal data will be requested or recorded during the interview. I want to record this interview so that we can analyse your answers for the research. The recording will be stored securely and deleted after we transcribe the interview anonymously. We will not request any personal data, but we will ask for some background information. The interview will be about your experience with scabies, what you did when you were diagnosed with scabies, and why. There are no right or wrong answers; we are interested in your personal opinion and experience.

*A few important points to mention before starting the interview*

I have/have not received the signed consent form. Are you in a quiet place where we can conduct the interview? How much time do you have? The incentive voucher will be sent by post. Can you email me your address? Do you have any questions before I start the interview?

Then I will start recording now.

**Background information**

We will start with some general questions about your background and living conditions.

- Would you like to name your gender for the recording?
- How old are you?
- In which city do you study?
- How do you live (for example, in a student house, with parents, in a studio): if living in a student house:
  - Is it a student association housing?
  - How many housemates do you have?
  - Do you have common areas? If yes, which? Do you use them (how often and for how long?)
- Are there any other apartments that you frequently visit (e.g. parents, girl/boyfiend)?
- Are you in a relationship?

**Experience with scabies**

Can you tell me something about your experience with scabies?

- How many times have you had scabies so far? When was the last time?
- How long did it last?
- How did you know that it was scabies?
- [If the student experienced uncertainty about their scabies status:] How did you deal with the uncertainty about your scabies status?
- How did you experience your scabies infection?
  - Negative/positive and why?
  - How did you deal with the negative aspects of scabies (if any)?
  - Did you experience many symptoms?
    - How did you deal with them?
    - Did the symptoms influence your sleep, daily life or study?
- What was your first reaction when you found out you have scabies?
  - Why did you react that way?
  - Which emotions and thoughts played a role in this reaction?
- Did people close to you know about your scabies infection?
  - If not, why did you not share this with others?
  - If yes, what reactions did you receive when disclosing your scabies status?
    - How did you deal with these reactions?
- Did you receive support from people close to you?
  - [If yes,] What kind of support and from whom? And how did you experience this support?
  - [If not,] Did you need support? Why did you feel that way? What kind of support would have helped you?

**Experience with contact notification (CN)**

Can you tell me something about your experience with CN?

- - To what extent have you notified others about your scabies status?
    - If no one, why not?
  - Which individuals did you notify?
    - Who were they (e.g., housemates, parents, friends, etc.)?
    - How did you know which individuals should be notified?
  - Why did you decide to notify someone (or not)?
  - When you found out you had scabies, did you immediately notify these people?
    - [If no,]
      - Why did you wait to tell them?
      - Which individuals were notified later?
      - Are there any possible consequences of not immediately notifying individuals? Can you name them?
        - [If not,] How does not immediately notifying affect the spread of scabies?
    - [If yes,] Why did you notify immediately, and which individuals were notified?
  - Is there any contact that you notified later? When was that, and why?
  - How did you decide which individuals should be notified?
    - Are you familiar with the national guidelines about scabies?
    - Did you notify your contacts on your own initiative, or did you receive any advice from others?
      - [If yes,] From whom, why and what was the advice?
  - Do you think that people close to you expect you to notify your contacts?
    - [If yes,] Who and why?
  - Are you confident that you have notified the right individuals and why?
  - How did you approach contact notification, and why did you choose that approach?
    - Did your approach differ for each type of contact? Why and how did it differ?
    - [If the student experienced scabies more than once:] Did your approach differ during each episode and why (not)? Why and how did it differ?
  - How did you experience CN?
    - What went well and what did not?
    - Was there anything difficult or easy, and why?
    - Did this experience differ for each type of contact?
    - How did you feel? And did these feelings differ for each type of contact?
  - How did the contacts react after CN?
    - How did you experience these reactions?
    - Did you receive any negative reactions that you didn’t like?
      - What were these reactions?
      - From whom?
      - How did you deal with it?
    - Did you feel any shame when telling others about your scabies experience?
  - What is your thought on CN?
    - Do you think CN is important/not important, useful/not useful, effective/not effective, etc. and why (not)
  - Do you feel that you needed support during CN?
    - [If so,] What kind of support did you need and from whom?
    - Did you have enough information to notify your contacts?
      - If not, what information do you need?
  - Did you provide your contacts with information during CN?
    - If not, why not?
    - If yes, what was that information? Why did you share that and how did you know about it?
    - Is the advice intended for the contacts clear to you?
    - Who is responsible for providing contacts with the correct information and advice on what to do and why?
  - To what extent do students notify their contacts? And why?
    - Why do you think that?
  - What is needed to ensure that students like you will notify all their contacts, in time, in the future?
  - Did you receive a CN yourself?
    - [If yes,]
      - When was that, and from whom?
      - Did you also get scabies after the CN?
      - How did you experience the CN?
    - [If not,]
      - How did you feel about not receiving a CN?
      - Would you have wanted to be notified? Why or why not?
  - If you were to get scabies again, would you handle the contact notifications in the same way?
    - Why or why not?
    - What would you do/not do, or what would you do differently and why?
  - If you had the option to notify your contacts anonymously, would you use it?
    - Why or why not?

We have come at the end of the interview.
Is there anything you would like to add or that I might be forgotten to ask?

Thank you very much for your participation!
